# Screening for thalassemia carriers among the Han population of childbearing age in Southwestern of China

**DOI:** 10.3389/fgene.2024.1356068

**Published:** 2024-04-10

**Authors:** Yepei Du, Cong Zhou, Jing Wang, Yanting Yang, Hui Liu

**Affiliations:** ^1^ Department of Medical Genetics/Prenatal Diagnostic Center, West China Second University Hospital, Sichuan University, Chengdu, China; ^2^ Key Laboratory of Birth Defects and Related Diseases of Women and Children (Sichuan University), Ministry of Education, Chengdu, China

**Keywords:** α-thalassemia, β-thalassemia, carrier screening, NGS, fluorescence PCR melting curve method

## Abstract

**Purpose:**

Thalassemia is a severe hereditary blood disorder that poses a significant threat to human health and leads to mortality and disability. It is one of the most prevalent monogenic diseases worldwide. The aim of this study was to analyze the molecular epidemiological data of individuals of childbearing age from the Han ethnic group with thalassemia in Southwest China and to explore the application of next-generation sequencing (NGS) technology in screening thalassemia carriers.

**Methods:**

The participants were Han males and females of childbearing age who sought medical advice at the West China Second University Hospital, Sichuan University from June 2022 to June 2023. We detected α- and β-thalassemia mutations using full-length capture of the thalassemia genes and NGS technology.

**Results:**

In a cohort of 1,093 participants, 130 thalassemia carriers were identified, with an overall detection rate of 11.89% (130/1,093). Among these, 0.91% (10/1,093) had mutations that could not be detected using traditional PCR techniques. The proportions of carriers with α-, β-, and α-complexed β-thalassemia gene mutations were 7.68% (84/1,093), 3.93% (43/1,093), and 0.27% (3/1,093), respectively. We identified a novel HBA2 c.166del variant that has not been previously reported.

**Conclusion:**

Using NGS technology, we found that the mutation-carrying rate of thalassemia genes was 11.89% in the Han population of childbearing age in Southwest China. Compared with the results of traditional PCR techniques, NGS detected an additional 0.91% (10/1,093) rare genetic variants. NGS technology should be utilized as the primary screening method for thalassemia carriers among Han nationality people of childbearing age in Southwest China.

## Introduction

Thalassemia, also known as Mediterranean anemia, is an autosomal recessive genetic disease and one of the most common monogenic genetic diseases worldwide (Taher et al., 2018). It belongs to the category of hereditary hemoglobinopathies. The most important forms of thalassemia are α-thalassemia and β-thalassemia, which are primarily caused by the imbalance of α-like and non-α-like globin chain production. This imbalance leads to a series of symptoms including red blood cell hemolysis, ineffective erythropoiesis, and iron overload (Kattamis et al., 2022). About 1.5% of the global population carry a β-thalassemic allele, and about 5% carry an α-thalassemic allele (Piel and Weatherall, 2014; Kattamis et al., 2020). Currently, the treatment of patients with thalassemia relies on supportive therapies such as blood transfusion and iron chelation. Only a few patients receive curative treatment, and allogeneic hematopoietic stem cell transplantation (allo-HSCT) remains the only curative method. However, allo-HSCT has not been widely implemented due to challenges in matching, high costs, and unsatisfactory outcomes (Rachmilewitz and Giardina, 2011; Kattamis et al., 2022). Therefore, premarital, pre-pregnancy, and prenatal thalassemia gene screening in areas with a high incidence of thalassemia is the most effective measure to avoid the birth of children with severe thalassemia (Ip and So, 2013).

Geographical heterogeneity is a prominent characteristic of thalassemia. Globally, thalassemia carriers are concentrated in the Mediterranean, the Middle East, Central Asia, India, and southern China (Colah et al., 2010). In China, thalassemia is prevalent in the southern regions, particularly in the Guangdong, Guangxi, Fujian, Yunnan, Guizhou, and Sichuan provinces (Huang et al., 2019). Southwestern China, including Chongqing, Sichuan, Guizhou and Yunnan provinces (He et al., 2017; Li et al., 2021; Tan et al., 2021; Li et al., 2022), has a high incidence of thalassemia. Mutations in the thalassemia genes are usually population-specific; each population has a unique spectrum of mutations (Williams and Weatherall, 2012). Currently, data on large samples of thalassemia carriers in the childbearing-age Han population in Southwest China are unavailable. Hence, it is crucial to explore the local epidemiological characteristics of thalassemia for effective targeted genetic detection and counseling.

At present, routine blood counts and indicators, such as mean corpuscular volume (MCV) and mean corpuscular hemoglobin (MCH), and hemoglobin component analysis (using high-performance liquid chromatography or capillary electrophoresis), are often used as first-line screening protocols for thalassemia (Ryan et al., 2010; Aliyeva et al., 2018). Although these are the fastest and most cost-effective screening protocols for thalassemia, they are susceptible to generating false negatives. Genetic testing is the gold standard for the diagnosis of thalassemia, identification of hemoglobin variants, clarification of complex cases, and prenatal diagnosis. Globally, gap-PCR and PCR-reverse dot blotting (PCR-RDB) are commonly used to diagnose thalassemia. However, the detection ranges of gap-PCR and PCR-RDB are limited; these methods only cover a small number of thalassemia gene mutation types (3 common deletion α-thalassemia mutations, 3 common non-deletion α-thalassemia mutations, and 17 common β-thalassemia mutations). Comparatively, next-generation sequencing (NGS) has significantly improved the detection range and accuracy and is able to detect more samples simultaneously. However, NGS has disadvantages in terms of detecting highly homologous regions, gene rearrangements, and large fragment deletions/duplications (Aliyeva et al., 2018). Although long-read third-generation sequencing has the advantage of detecting copy number variations (CNVs) and mutations in homologous genes (Hassan et al., 2023), it is not widely used in clinical practice because of its high cost. Considering the possible limitations of NGS technology in detecting homologous regions of *HBA1* and *HBA2*, we utilized NGS and fluorescence PCR melting curve method to test all participants. Additionally, Sanger sequencing, quantitative Real-time polymerase chain reaction (qRT-PCR), and PCR-electrophoresis were employed to validate the point mutations and α-thalassemia triplications detected from the NGS results. In this study, we aimed to analyze the molecular epidemiological characteristics of thalassemia among the childbearing-age Han population in Southwest China to provide a reference for prenatal screening, prenatal diagnosis, and birth defect prevention and control. We also explored the application of NGS for the detection of thalassemia.

## Materials and methods

### Participants

A total of 1,093 Han males and females of childbearing age who sought medical advice at the West China Second University Hospital, Sichuan University, from June 2022 to June 2023 were recruited for the study. The participants were mainly from various provinces in Southwest China. This study was approved by the Medical Ethics Committee of the West China Second University Hospital of Sichuan University (20200052gc). All experiments were performed in accordance with relevant guidelines and regulations. All participants received comprehensive genetic counseling and provided informed consent.

### NGS

Genomic DNA was extracted from whole blood samples using nucleic acid extraction kit (magnetic bead method) (MyGenostics, Chongqing, China). The extracted DNA was required to have a concentration ≥25 ng/μL and a 260/280 ratio between 1.7 and 2.0. Library preparation was performed using an enzyme digestion method with a V5.0 library preparation kit (MyGenostics). Library products were required to have a fragment length distribution of 200–500 bp. Then 8–12 samples were mixed into one hybridization unit with 600 ng every sample. Added probes and buffer from the GenCap^®^ universal nucleic acid fragment enrichment purification kit V2.3 (MyGenostics) to each hybridization unit. And hybridized for 12–24 h to Hybridization enrichment. The probes are double-stranded DNA, which covering the full length of the *HBA1, HBA2,* and *HBB* genes. The concentration of each hybridization unit was detected by absolute quantitative QPCR, and pooling was performed based on the QPCR results. Sequencing was performed on the Illumina NextSeq 500 platform (Illumina, San Diego, CA, USA) using the NextSeq CN500 Middle Output Kit (Illumina). After sequencing, the data were aligned to preprocessed data and human reference genome GRCh37/hg19 using BWA (version 0.7.10; https://www.plob.org/tag/bwa). GATK (version 4.0.8.1; https://www.broadinstitute.org/gatk/) was used for base recalibration, and the Single Nucleotide Polymorphisms (SNPs) and Insertions and Deletions (InDels) results were annotated using the ANNOVAR software (version 1; http://annovar.openbioinformatics.org/en/latest/) (McCombie et al., 2019). Pathogenicity analysis was performed according to the sequence variation interpretation standards and guidelines recommended by the American College of Medical Genetics and Genomics and Association for Molecular Pathology (Richards et al., 2015).

### Validation experiments

All DNA samples were tested for three common deletion α-thalassemia mutations in the Chinese population, including Southeast Asian deletion type (--^SEA^/), left deletion type (-α^4.2^/), and right deletion type (-α^3.7^/). DNA was tested using fluorescent PCR melting curve method (deletion α-thalassemia kit, Zeesan, Xiamen, China) on the cobas z 480 analyzer (Roche, Switzerland) platform. Samples with NGS results of *HBA1, HBA2* or *HBB* gene mutations (such as SNPs and InDels) were validated using Sanger sequencing. Subsequent to performing primer design and PCR, Sanger sequencing was carried out, and the sequencing results were analyzed using Chroms software (version 2.4.1; Biosoft).

Samples showing increased copy numbers of *HBA1/HBA2* (unkown samples) based on NGS analysis results were subjected to qRT-PCR and the 2^−ΔΔCt^ method (Livak and Schmittgen, 2001). There are three groups of nucleotide fragments with high homology in *HBA1* and *HBA2*, labeled as X segment, Y segment, and Z segment (Farashi and Harteveld, 2018). According to the molecular structure of ααα^anti3.7^, ααα^anti4.2^, and HKαα, we measured the copy number of Z segments to detect the copy numbers of *HBA1/HBA2* (Long and Liu, 2021). Primers were designed for exon 3 of *HBA2* in Z segments, and the β-actin gene was selected as the internal control gene. We ensure that the products of each pair of primers have unique sequences. We selected a sample with two copy numbers of *HBA1/HBA2* as calibrator sample. The threshold cycle (Ct) were detected on a 7500 FAST Dx Real-Time PCR platform (Thermo Fisher Scientific Inc, Waltham, MA, USA). All sample performed experiments three times. We calculated the ΔCt value of the unkown samples and calibrator sample tested as bellow: ΔCt = Ct _*HBA2*_ -Ct_β-actin_. And we calculated ΔΔCt value of each unkown sample as bellow: ΔΔCt _unkown sample_ = ΔCt_unkown sample_- ΔCt_calibrator sample_. Using the 2^−ΔΔCt^ method to calculate the relative quantitative value, the result of 2^−ΔΔCt^ is the relative ratio of the unkown samples and calibrator sample. In addition, we can get the 2^−ΔΔCt^ value of unkown samples on 7500 FAST Dx Real-Time PCR platform directly. And the genotype was verified using the PCR-electrophoresis (6 α-thalassemia gene detection kit, Yaneng, Shenzhen, China). We presented several representative results of qRT-PCR and PCR-electrophoresis (Supplementary Figure S1). All samples which were identified thalassemia carriers with NGS were validated using another method. The primers information is shown in Supplementary Table S1.

## Results

Of the 1,093 participants tested in this study, 130 thalassemia carriers were identified, with an overall detection rate of 11.89% (130/1,093). From amongst the 1,093 participants, 84 (7.68%) cases of α-thalassemia, 43 (3.93%) cases of β-thalassemia, and 3 (0.27%) cases of α-complex β-thalassemia were detected. We detected 25 participants with α-globin gene triplications in the total study population, with a detection rate of 2.28% (25/1,093). A total of 152 thalassemia and α-globin gene triplications carriers were detected, mainly from Sichuan, Xizang and Chongqing provinces.

Seven α-thalassemia genotypes were identified, among which the deletion α-thalassemia genotypes were the most common, with 79 cases accounting for 94.04% (79/84) of all α-thalassemia cases. The most common deletion α-thalassemia genotype was --^SEA^/αα, which accounted for 48.80% (41/84) of α-thalassemia cases. The most common non-deletion α-thalassemia genotype was HBA2 c.369C>G heterozygous mutation, accounting for 2.38% (2/84) of α-thalassemia cases. We identified sixteen β-thalassemia genotypes, five of which were hemoglobin variants. The most common genotype was HBB c.126-129del heterozygous mutation, accounting for 23.25% (10/43) of β-thalassemia cases. Three cases of α-complex β-thalassemia were identified, with three different genotypes. Further details are listed in Table 1.

**TABLE 1:** The thalassemia screening results of the 1,093 participants.

HGVS^a^ nomenclature	Genotype	Number	Frequency (%)	Phenotype
α-thalassemia				
NC_000016. 9: g. 215400_234700del	--^SEA^/αα	41	48.80	α^0^/αα
NC_000016. 9: g. 223300_227103del	-α^3.7^/αα	32	38.09	αα^+^/αα
NC_000016. 9: g. 219817_(223755_ 224074)del	-α^4.2^/αα	6	7.14	αα^+^/αα
HBA2 c. 369C>G	Hb Westmead	2	2.38	αα^T^/αα
HBA2 c. 166del	—	1	1.19	αα^T^/αα
HBA2 c. 272_279del	—	1	1.19	αα^T^/αα
HBA2 c. 427T>C	Hb Constant Spring	1	1.19	αα^T^/αα
Total	—	84	100	—
β-thalassemia				
HBB c. 126_129delCTTT	Codons 41/42 (-TTCT)	10	23.25	β^0^/β^N^
HBB c. 52A>T	Codon 17 (A>T)	9	20.93	β^0^/β^N^
HBB c. 316-197C>T	IVS-Ⅱ-654 (C>T)	7	16.27	β^+^/β^N^
HBB c. -78A>G	−28 (A>G)	3	6.97	β^+^/β^N^
HBB c. -79A>G	−29 (A>G)	2	4.65	β^+^/β^N^
*HBB* del of exons 1-3	—	2	4.65	β^0^/β^N^
HBB c. *110T>C	Poly A (T>C)	1	2.32	β^+^/β^N^
HBB c. -100G>A	−50 (G>A)	1	2.32	β^+^/β^N^
HBB c. 130G>T	Codon 43 (G>T)	1	2.32	β^0^/β^N^
HBB c. 216_217insA	Codons 71/72 (+A)	1	2.32	β^0^/β^N^
HBB c. 84_85insC	Codons 27/28 (+C)	1	2.32	β^0^/β^N^
HBB c. 180G>C	Hb J-Lome ^b^	1	2.32	Hb J-Lome ^b^
HBB c. 341T>A	Hb^b^ New York	1	2.32	Hb^b^ New York
HBB c. 68A>C	Hb^b^ G-Coushatta	1	2.32	Hb^b^ G-Coushatta
HBB c. 68A>G	Hb^b^ G-Taipei	1	2.32	Hb^b^ G-Taipei
HBB c. 79G>A	Hb^b^ E	1	2.32	Hb^b^ E
Total	—	43	100	—
The coexistence of α-thalassemia and β-thalassemia				
**—**	- -^SEA^/αα; HBB c. 126_129del	1	33.33	α^0^/αα; β^0^/β^N^
**—**	--^SEA^/αα; HBB c. 316-197C>T	1	33.33	α^0^/αα; β^+^/β^N^
**—**	-α^4. 2^/αα; HBB c. 216_217insA	1	33.33	αα^+^/αα; β^0^/β^N^
Total	—	3	100	—

^a^ HGVS, the Human Genome Variation.

^b^ Hb, Hemoglobin.

**FIGURE 1:**
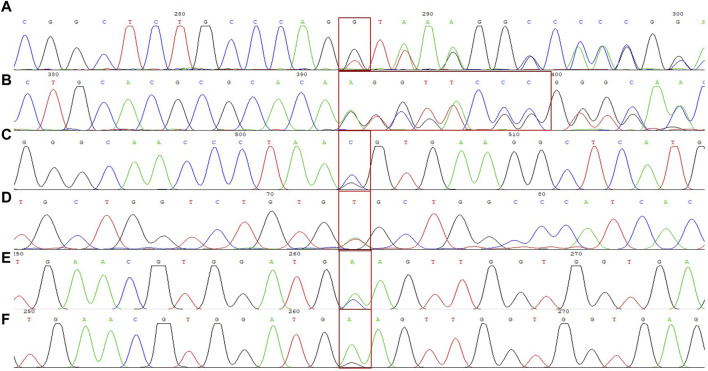
DNA sequencing results of 6 rare forms of thalassemia. **(A)** HBA2 c. 166del; **(B)** HBA2 c. 272_279delAGCTTCGG; **(C)** HBB c. 180G>C; **(D)** HBB c. 341T>A; **(E)** HBB c. 68A>C; **(F)** HBB c. 68A>G.

**FIGURE 2:**
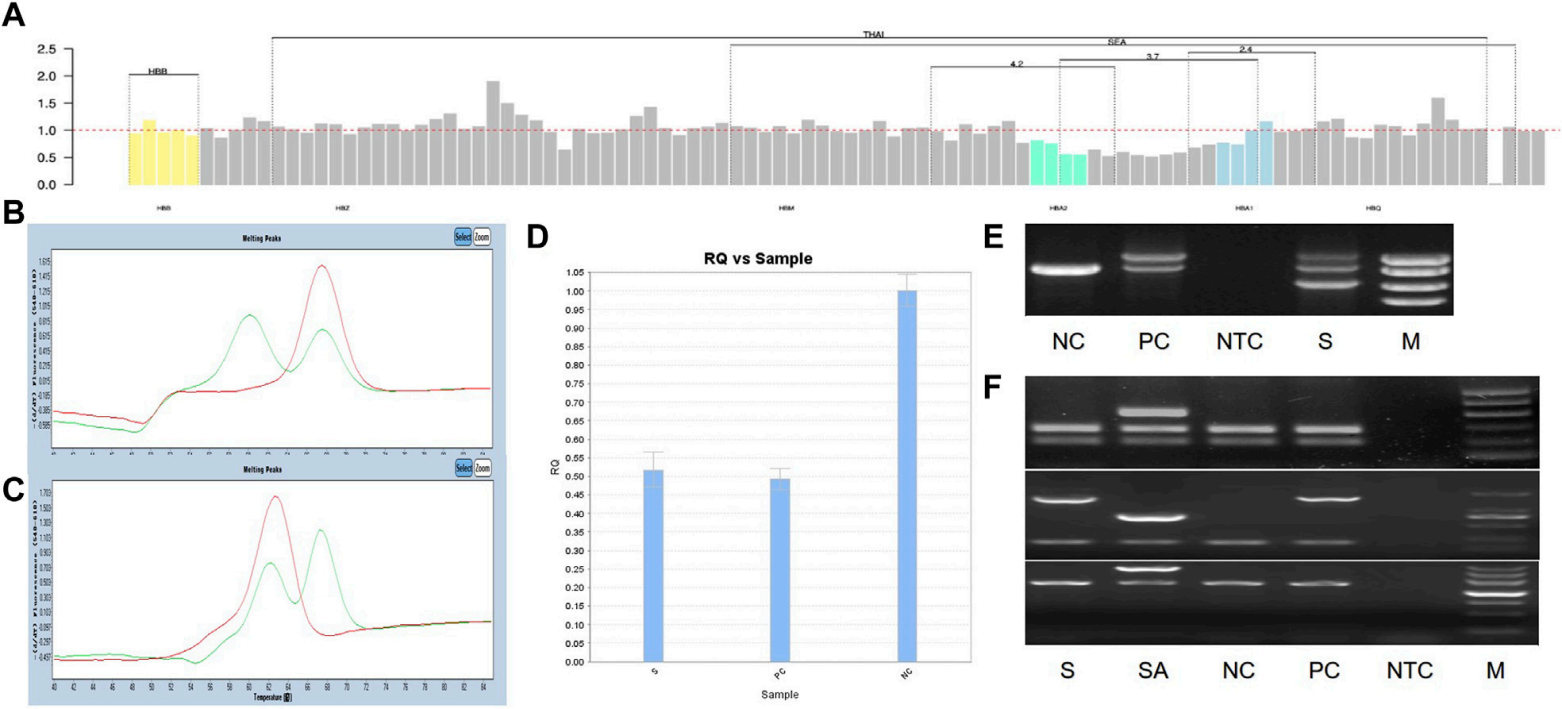
All experimental results of 1 sample with genotype HKαα/-α^4.2^, S is sample, PC is positive control, NC is normal control, NTC is No template control, M is marker. **(A)** The result of NGS; **(B, C)** The result of Fluorescent PCR melting curve method, **(B)** A-ROX, **(C)** B-ROX, the green peak pattern is sample, the red peak pattern is normal control; **(D)** The result of qRT-PCR, PC is --^SEA^/αα; **(E)** The result of gap-PCR, PC is -α^3.7^/αα; **(F)** The result of PCR-electrophoresis, SA is Quality control of reagents, PC is HKαα/αα.

We identified a novel mutation (HBA2 c.166del, p.Val56LeufsTer12). To the best of our knowledge, the mutation has not been recorded in ClinVar, the Human Gene Mutation Database or PubMed database, and it has not been reported in literature. According to the prediction, the mutation may lead to premature termination of the codons. Beyond that, we identified six rare thalassemia genotypes: HBA2 c.272_279delAGCTTCGG heterozygote, HBB c.180G>C heterozygote, HBB c.341T>A heterozygote, HBB c.68A>C heterozygote, HBB c.68A>G heterozygote, and heterozygous deletion of *HBB* exons 1-3. Heterozygous deletion of *HBB* exon 1-3 was verified using multiplex ligation-dependent probe amplification, whereas the remaining rare thalassemia genotypes were verified using Sanger sequencing (Figure 1). NGS results of 25 samples indicated increased copy numbers of *HBA1/HBA2,* which were verified using qRT-PCR and PCR-electrophoresis. From the 25 samples, six different genotypes were detected (Table 2). The most common genotype was ααα^anti3.7^/αα, accounting for 48% (12/25) of all α-globin gene triplication cases. One genotype was more cryptic than others and required several analyses. A case was identified as -α^3.7^/αα using NGS. The fluorescence PCR melting curve method showed the presence of both wild-type and deletion peaks in the A-ROX and B-ROX channels, indicating the simultaneous presence of heterozygous deletions of -α^3.7^ and -α^4.2^. However, the result did not correspond to the -α^3.7^/-α^4.2^ peak pattern. The electrophoresis result of gap-PCR showed the simultaneous presence of -α^3.7^/, -α^4.2^/, and a normal band. The qRT-PCR results indicated that the copy number of *HBA1/HBA2* was 1. The result of PCR-electrophoresis showed that there was a positive HKαα band and a normal band. Taking all the results into account, we identified this genotype as HKαα/-α^4.2^ (Figure 2). We identified seven couples with a high-risk of thalassemia in this study (Supplementary Table S2).

**TABLE 2:** The α-Globin gene triplication results of the 1,093 participants.

NGS^a^	Fluorescence PCR melting curve method	qRT-PCR^b^ (copy number)	PCR-electrophoresis	Possible genotype	Number	Frequency (%)
ααα^anti3.7^	Normal	3	ααα^anti3.7^	ααα^anti3.7^/αα	12	48
ααα^anti4.2^	Normal	3	ααα^anti4.2^	ααα^anti4.2^/αα	9	36
- α^3.7^+ααα^anti4.2^	- α^3.7^	2	HKαα	HKαα/αα	1	4
αααα^anti3.7^	Normal	4	ααα^anti3.7^	αααα^anti3.7^/αα	1	4
- -^SEA^+ααα^anti3.7^	- -^SEA^	2	ααα^anti3.7^	--^SEA^/ααα^anti3.7^	1	4
-α^3.7^	- α^3.7^+- α^4.2^	1	HKαα	HKαα/-α^4.2^	1	4
Total	—	—	—	—	25	100

^a^ NGS, next-generation sequencing.

^b^ qRT-PCR, quantitative Real-time polymerase chain reaction.

## Discussion

In this study, the carrier rate of α-thalassemia was higher than that of β-thalassemia in Southwest China. Secondly, the mutations of α-thalassemia carriers were mainly --^SEA^/αα and -α^3.7^/αα, while β-thalassemia were mainly codon 41/42 (-TTCT), codon 17 (A>T), and IVS-II-654 (C>T). These two findings mentioned above in our study are consistent with previous reports from various regions of China (He et al., 2017; Zhuang et al., 2020; Li et al., 2021; Tan et al., 2021; Li et al., 2022; Wei et al., 2022; Xian et al., 2022; Yu et al., 2022; Xi et al., 2023; Yang et al., 2023) (Table 3). Li et al. reported that the thalassemia carrying rate in Sichuan region was 2.6%, which was lower than this study. This could be due to Li et al.’s study included a total of 42155 participants, with only 2430 hematologically positive individuals undergoing molecular diagnosis (Li et al., 2021). Baise, Guangxi is a multi-ethnic area with a high incidence of thalassemia genes in the population, and the carrier rate of thalassemia of Baise is higher than the results of this study (Wei et al., 2022). The participants in Hainan, Guangdong and Fujian (Quanzhou) had positive hematological results and belonged to high-risk populations, resulting in a much higher carrier rate of thalassemia than that in this study. The thalassemia carrier rate in our study was higher than that reported in Hunan (Xi et al., 2023). Nevertheless, the carrier rate of thalassemia in cities in Hunan varies according to the geographical location. Although the data in this study were similar to those from Chongqing, Guizhou (Qianxinan) and Jiangxi (Ganzhou), there were slight differences in the characteristics of the mutation spectra. This study identified a novel mutation (HBA2 c.166del), which has not been previously reported. In addition, this study identified six rare thalassemia gene mutations. Among them, HBA2 c.272_279delAGCTTCGG was first reported in Guangxi Zhuang Autonomous Region, China, and the phenotype of HBA2 c.272_279delAGCTTCGG heterozygote was speculated to be similar to -α^3.7^/αα, showing static thalassemia (Li et al., 2019). According to the Database of Human Hemoglobin Variants and Thalassemia Mutations, all individuals with heterozygous mutations in HBB c.180G>C, c.341T>A, c.68A>C, and c.68A>G present normally. Exon 1-3 deletion in *HBB* has been reported in foreign countries (Hogan et al., 2018), but there is no report in China. In the two cases of *HBB* exons 1-3 deletion heterozygosity in this study, hematological tests showed normal levels of Hemoglobin (HGB), decreased MCV or MCH, and elevated Fetal hemoglobin.

**TABLE 3:** Comparison of thalassemia mutations in different regions of Southern China.

Region	α-thalassemia Frequency (%)	β-thalassemia Frequency (%)	Complex thalassemia Frequency (%)	Genetic detection methods	Notes	References
Sichuan	1.83	0.75	0.05	Gap-PCR	Only parts of the participants who had positive hematological results had molecular diagnosis results	Li et al. (2021)
				RDB^a^		
Chongqing	6.16	4.13	0.12	PCR-flow fluorescence hybridization	—	Li et al. (2022)
Hainan	59.22	3.76	7.57	Gap-PCR	The participants had RBC^b^ results of MCV^c^<82 fL or MCH^d^ <27 pg	Yu et al. (2022)
				RDB^a^		
Yunnan	44.1	20.0	—	NGS^e^	The participants were Dai nationality in Yunnan	He et al. (2017)
				Sanger sequencing		
Hunan	4.27	1.79	0.09	NGS^e^	—	Xi et al. (2023)
Guangdong	15.07	3.80	0.61	Gap-PCR	Only for those were highly suspicious of thalassemia carriers	Xian et al. (2022)
				RDB^a^		
Baise, Guangxi	18.57	7.50	1.88	Gap-PCR	—	Wei et al. (2022)
				RDB^a^		
				AS-PCR		
Qianxinan, Guizhou	8.91	3.36	0.63	NGS^e^	—	Tan et al. (2021)
				Gap-PCR		
Ganzhou, Jiangxi	10.48	3.61	0.44	NGS^e^	—	Yang et al. (2023)
				Gap-PCR		
Quanzhou, Fujian	28.27	12.06	0.78	Gap-PCR	The participants included individuals with positive hematological testing results, with a family history, or whose spouses were thalassemia carriers	Zhuang et al. (2020)
				RDB^a^		
This Study	7.68	3.93	0.27	NGS^e^	—	—
				Fluorescence PCR melting curve method		

^a^ RDB, PCR-reverse dot blotting.

^b^ RBC, routine blood counts.

^c^ MCV, mean corpuscular volume.

^d^ MCH, mean corpuscular hemoglobin.

^e^ NGS, next-generation sequencing.

The α-thalassemia triplication or quadruplication rate in this study is 2.28%, which is close to Hunan Province of 1.99% (Xi et al., 2023). Of the 25 α-thalassemia triplication carriers, 15 did not take hematological testing (including two with --^SEA^/ααα^anti3.7^ and HKαα/-α^4.2^, respectively), nine returned normal results in routine blood counts or hemoglobin electrophoresis, and one showed mild anemia with 105 g/L HGB, 63.7 fL MCV, and 18.8 pg MCH (pre-pregnancy). The NGS result of a mild anemia participant was ααα^anti4.2^/αα complex HBB c.316-197C>T heterozygous, and her hemoglobin continued to decrease during pregnancy. At 29^+1^ weeks of pregnancy, routine blood counts revealed an HGB level of 87 g/L, indicating moderate anemia. In conclusion, patients with β-thalassemia combined with α-thalassemia triplication probably have aggravated clinical phenotype. And pregnancy may worsen the severity of anemia and increase the risk of anemia-related obstetric comorbidities and complications. Therefore, it is important to perform thalassemia gene testing in women during preconception.In this study, seven high-risk couples were identified. The women in the F03, F05, and F07 families in this study were pregnant. After genetic counseling, all three families chose to undergo amniocentesis for the prenatal diagnosis of thalassemia.

Currently, hematological testing (combined routine blood counts and hemoglobin component analysis) is commonly used as the first-line screening method for thalassemia in clinical practice (Ryan et al., 2010; Aliyeva et al., 2018). However, this method is prone to false negatives in thalassemia carriers with negative hematological test results. In this study, the false-negative rate for only using routine blood count examinations or hemoglobin electrophoresis was 30.61% (15/49) and 56.41% (22/39), respectively. When both two methods were used, the false-negative rate was 19.35% (6/31) (Table 4). The main methods used for the detection of thalassemia genes in clinical practice include Southern blot hybridization, high-resolution melting curve analysis, array-based detection, amplification refractory mutation system PCR (ARMS-PCR), real-time PCR, gap-PCR, and PCR-RDB (Vrettou et al., 2003; Chan et al., 2004; Huang et al., 2017; Hassan et al., 2023). Southern blot hybridization is considered the gold standard for large fragment deletion detection, especially for the α-globin gene. However, it requires a large sample volume and involves complex procedures, limiting its clinical application. High-resolution melting curve analysis, array-based detection, ARMS-PCR, real-time PCR, gap-PCR, and PCR-RDB have limited detection ranges and can only detect known thalassemia gene variants. Gap-PCR and PCR-RDB are the most commonly used methods for diagnosing point mutations of thalassemia genes and deletions of α-globin genes globally. However, when using only gap-PCR combined with PCR-RDB, the detection rate of thalassemia was 10.97% (120/1,093) in our study. Two samples carrying mutations of α-globin genes (HBA2 c.166del and c.272_279del) and eight samples carrying mutations of β-globin gene (*HBB* exons 1-3del, c.*110T>C, c.-100G>A, c.180G>C, c.341T>A, c.68A>C, and c.68A>G) could not be detected.

**TABLE 4:** Sensitivity and specificity of HbA2^a^ and MCV^b^+MCH^c^ levels for thalassaemia carriers screened by NGS^d^.

Methods	True positive	False positive	True negative	False negative	Sensitivity (%)	Specificity (%)	PPV (%)	NPV (%)
MM	34	23	385	15	69.38	94.36	59.64	96.25
HbA2	17	14	235	22	43.58	94.37	54.83	91.43
HbA2+MM^e^	25	20	196	6	80.64	90.74	55.55	97.02

^a^ HbA2, Hemoglobin A2.

^b^ MCV, mean corpuscular volume.

^c^ MCH, mean corpuscular hemoglobin.

^d^ NGS, next-generation sequencing.

^e^ MM, MCV+MCH.

The thalassemia genes *HBA1* and *HBA2* detected in this study are highly homologous, and NGS has certain limitations (Aliyeva et al., 2018). The capture probe used in this study was a double-stranded DNA probe covering the full lengths of the *HBA1, HBA2,* and *HBB* genes, which could stably capture target genes with good repeatability. Probe density increased locally in areas with common deletions and duplications. Simultaneously, the probe density and length were adjusted according to the GC content and melting temperature value for special areas to ensure a higher capture efficiency and more accurate CNVs analysis. Our results showed that the NGS results of α-thalassemia carriers were consistent with the corresponding validation experiments. Based on these results, detection using full-length capture of the thalassemia genes and NGS technology are accurate and reliable. In this study, the NGS results of one sample indicated that the copy number of *HBA1/HBA2* was four, but the genotype could not be distinguished as αααα^anti3.7^/αα or ααα^anti3.7^/ααα^anti3.7^. PCR-electrophoresis confirmed the genotype to be αααα^anti3.7^/αα. Therefore, NGS can detect an increase in the copy number of genes but cannot accurately assess the fertility risk because of the inability to identify complex rearrangement genotypes. Although this situation is very rare, if clinical testing shows that the genotype cannot be determined, other methods, such as PCR electrophoresis or third-generation sequencing, still need to be used for further validation.

## Conclusion

In summary, there is a high incidence of thalassemia among people of childbearing age in Southwest China. Our results confirmed that NGS technology can serve as an economical, rapid, high-throughput, and efficient strategy for screening thalassemia carriers.

## Data Availability

All datasets generated for this study are included in the article/Supplementary Material. Further inquiries can be directed to the corresponding authors.

## Abbreviations

NGS, next-generation sequencing; allo-HSCT, allogeneic hematopoietic stem cell transplantation; MCV, mean corpuscular volume; MCH, mean corpuscular hemoglobin; PCR-RDB, PCR-reverse dot blotting; CNVs, copy number variations; qRT-PCR, quantitative Real-time polymerase chain reaction; SNPs, Single Nucleotide Polymorphisms; InDels, Insertions and Deletions; Ct, threshold cycle; ARMS-PCR, amplification refractory mutation system PCR; HGB/Hb, Hemoglobin.

## References

[B1] Aliyeva G. Asadov C. Mammadova T. Gafarova S. Abdulalimov E. (2018). Thalassemia in the laboratory: pearls, pitfalls, and promises. Clin. Chem. Lab. Med. 57 (2), 165–174. 10.1515/cclm-2018-0647 30138112

[B2] Chan K. Wong M. S. Chan T. K. Chan V. (2004). A thalassaemia array for Southeast Asia. Br. J. Haematol. 124 (2), 232–239. 10.1046/j.1365-2141.2003.04758.x 14687035

[B3] Colah R. Gorakshakar A. Nadkarni A. (2010). Global burden, distribution and prevention of β-thalassemias and hemoglobin E disorders. Expert Rev. Hematol. 3 (1), 103–117. 10.1586/ehm.09.74 21082937

[B4] Farashi S. Harteveld C. L. (2018). Molecular basis of α-thalassemia. Blood cells, Mol. Dis. 70, 43–53. 10.1016/j.bcmd.2017.09.004 29032940

[B5] Hassan S. Bahar R. Johan M. F. Mohamed Hashim E. K. Abdullah W. Z. Esa E. (2023). Next-generation sequencing (NGS) and third-generation sequencing (TGS) for the diagnosis of thalassemia. Diagn. (Basel) 13 (3), 373. 10.3390/diagnostics13030373 PMC9914462 36766477

[B6] He J. Song W. Yang J. Lu S. Yuan Y. Guo J. (2017). Next-generation sequencing improves thalassemia carrier screening among premarital adults in a high prevalence population: the Dai nationality, China. Genet. Med. 19 (9), 1022–1031. 10.1038/gim.2016.218 28125089

[B7] Hogan G. J. Vysotskaia V. S. Beauchamp K. A. Seisenberger S. Grauman P. V. Haas K. R. (2018). Validation of an expanded carrier screen that optimizes sensitivity via full-exon sequencing and panel-wide copy number variant identification. Clin. Chem. 64 (7), 1063–1073. 10.1373/clinchem.2018.286823 29760218

[B8] Huang H. Xu L. Chen M. Lin N. Xue H. Chen L. (2019). Molecular characterization of thalassemia and hemoglobinopathy in Southeastern China. Sci. Rep. 9 (1), 3493. 10.1038/s41598-019-40089-5 30837609 PMC6400947

[B9] Huang Q. Wang X. Tang N. Yan T. Chen P. Li Q. (2017). Simultaneous genotyping of α-thalassemia deletional and nondeletional mutations by real-time PCR-based multicolor melting curve analysis. J. Mol. Diagn 19 (4), 567–574. 10.1016/j.jmoldx.2017.04.003 28506685

[B10] Ip H. W. So C. C. (2013). Diagnosis and prevention of thalassemia. Crit. Rev. Clin. Lab. Sci. 50 (6), 125–141. 10.3109/10408363.2013.847236 24295057

[B11] Kattamis A. Forni G. L. Aydinok Y. Viprakasit V. (2020). Changing patterns in the epidemiology of β-thalassemia. Eur. J. Haematol. 105 (6), 692–703. 10.1111/ejh.13512 32886826 PMC7692954

[B12] Kattamis A. Kwiatkowski J. L. Aydinok Y. (2022). Thalassaemia. Lancet 399 (10343), 2310–2324. 10.1016/S0140-6736(22)00536-0 35691301

[B13] Li B. Han X. Ma J. Yang D. (2021). Mutation spectrum and erythrocyte indices characterisation of α-thalassaemia and β-thalassaemia in Sichuan women in China: a thalassaemia screening survey of 42 155 women. J. Clin. Pathol. 74 (3), 182–186. 10.1136/jclinpath-2020-206588 32719015

[B14] Li S. Y. Li Q. H. Yi S. W. Li C. L. (2022). Zhongguo shi yan xue ye xue za zhi. J. Exp. Hematol. 30 (1), 211–216. 10.19746/j.cnki.issn.1009-2137.2022.01.035 35123629

[B15] Li Y. Liang L. Tian M. Qin T. Wu X. (2019). Detection of Hb H disease caused by a novel mutation and --(SEA) deletion using capillary electrophoresis. J. Clin. Lab. Anal. 33 (7), e22949. 10.1002/jcla.22949 31199523 PMC6757179

[B16] Livak K. J. Schmittgen T. D. (2001). Analysis of relative gene expression data using real-time quantitative PCR and the 2(-Delta Delta C(T)) Method. Methods (San Diego, Calif.) 25 (4), 402–408. 10.1006/meth.2001.1262 11846609

[B17] Long J. Liu E. (2021). The carriage rates of αααanti3.7, αααanti4.2, and HKαα in the population of Guangxi, China measured using a rapid detection qPCR system to determine CNV in the α-globin gene cluster. Gene 768, 145296. 10.1016/j.gene.2020.145296 33181251

[B18] McCombie W. R. McPherson J. D. Mardis E. R. (2019). Next-generation sequencing technologies. Cold Spring Harb. Perspect. Med. 9 (11), a036798. 10.1101/cshperspect.a036798 30478097 PMC6824406

[B19] Piel F. B. Weatherall D. J. (2014). The α-thalassemias. N. Engl. J. Med. 371 (20), 1908–1916. 10.1056/NEJMra1404415 25390741

[B20] Rachmilewitz E. A. Giardina P. J. (2011). How I treat thalassemia. Blood 118 (13), 3479–3488. 10.1182/blood-2010-08-300335 21813448

[B21] Richards S. Aziz N. Bale S. Bick D. Das S. Gastier-Foster J. (2015). Standards and guidelines for the interpretation of sequence variants: a joint consensus recommendation of the American College of medical genetics and Genomics and the association for molecular Pathology. Genet. Med. 17 (5), 405–424. 10.1038/gim.2015.30 25741868 PMC4544753

[B22] Ryan K. Bain B. J. Worthington D. James J. Plews D. Mason A. (2010). Significant haemoglobinopathies: guidelines for screening and diagnosis. Br. J. Haematol. 149 (1), 35–49. 10.1111/j.1365-2141.2009.08054.x 20067565

[B23] Taher A. T. Weatherall D. J. Cappellini M. D. (2018). Thalassaemia. Lancet 391 (10116), 155–167. 10.1016/S0140-6736(17)31822-6 28774421

[B24] Tan M. Bai Y. Zhang X. Sun J. Huang C. Tian R. (2021). Early genetic screening uncovered a high prevalence of thalassemia among 18 309 neonates in Guizhou, China. Clin. Genet. 99 (5), 704–712. 10.1111/cge.13923 33439495

[B25] Vrettou C. Traeger-Synodinos J. Tzetis M. Malamis G. Kanavakis E. (2003). Rapid screening of multiple beta-globin gene mutations by real-time PCR on the LightCycler: application to carrier screening and prenatal diagnosis of thalassemia syndromes. Clin. Chem. 49 (5), 769–776. 10.1373/49.5.769 12709368

[B26] Wei B. Zhou W. Peng M. Long J. Wen W. (2022). The population incidence of thalassemia gene variants in Baise, Guangxi, P. R. China, based on random samples. Hematol. Amst. Neth. 27 (1), 1026–1031. 10.1080/16078454.2022.2119736 36066284

[B27] Williams T. N. Weatherall D. J. (2012). World distribution, population genetics, and health burden of the hemoglobinopathies. Cold Spring Harb. Perspect. Med. 2 (9), a011692. 10.1101/cshperspect.a011692 22951448 PMC3426822

[B28] Xi H. Liu Q. Xie D. H. Zhou X. Tang W. L. Tang G. (2023). Epidemiological survey of hemoglobinopathies based on next-generation sequencing platform in hunan province, China. Biomed. Environ. Sci. 36 (2), 127–134. 10.3967/bes2023.016 36861191

[B29] Xian J. Wang Y. He J. Li S. He W. Ma X. (2022). Molecular epidemiology and hematologic characterization of thalassemia in Guangdong province, southern China. Clin. Appl. Thromb. Hemost. 28, 10760296221119807. 10.1177/10760296221119807 35979587 PMC9393661

[B30] Yang T. Luo X. Liu Y. Lin M. Zhao Q. Zhang W. (2023). Next-generation sequencing analysis of the molecular spectrum of thalassemia in Southern Jiangxi, China. Hum. Genomics 17 (1), 77. 10.1186/s40246-023-00520-5 37592328 PMC10436446

[B31] Yu Y. Lu C. Gao Y. Li C. Li D. Wang J. (2022). Molecular spectrum, ethnic and geographical distribution of thalassemia in the southern area of hainan, China. Front. Pediatr. 10, 894444. 10.3389/fped.2022.894444 35783323 PMC9245522

[B32] Zhuang J. Jiang Y. Wang Y. Zheng Y. Zhuang Q. Wang J. (2020). Molecular analysis of α-thalassemia and β-thalassemia in quanzhou region Southeast China. J. Clin. Pathol. 73 (5), 278–282. 10.1136/jclinpath-2019-206179 31653757

