# Supplementary material for: Screening for thalassemia carriers among the Han population of childbearing age in Southwestern of China
Source: Front Genet. 2024 Apr 10;15:1356068. doi: 10.3389/fgene.2024.1356068 (PMC11039874; doi:10.3389/fgene.2024.1356068)
Supplement: Supplementary file 1 [file Table1.DOCX]

Supplementary Material

**FIGURE S1** Several representative results of qRT-PCR and PCR-electrophoresis


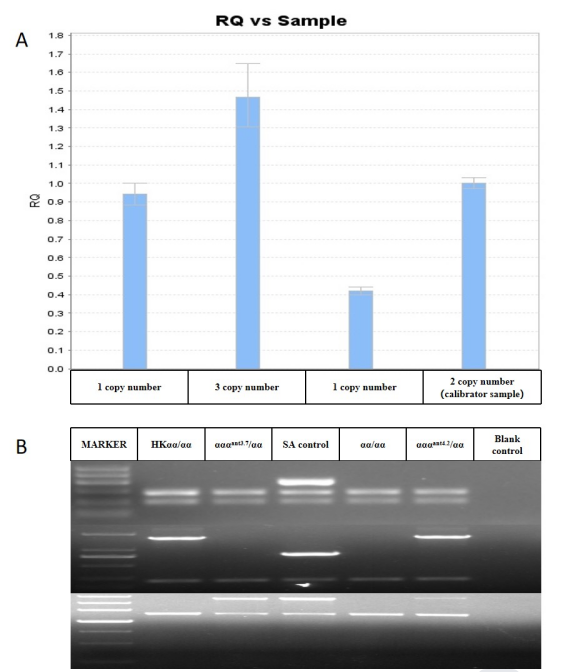


**FIGURE S1** Several representative results of qRT-PCR and PCR-electrophoresis. (A) The results of qRT-PCR and the 2^-ΔΔCt^ method; (B) The results of PCR-electrophoresis.

**TABLE S1** Primers for Sanger sequencing and qRT-PCR^a^

| **Method** | **Gene** | **Mutation** | **5’→3’ sequence** |
| --- | --- | --- | --- |
| **Sanger sequencing** | *HBB* | c. *110T>C | F: TTTGCAGCCTCACCTTCTTT  R: AAGCTCGCTTTCTTGCTGTC |
|  |  | c. 341T>A | F: TGGACTTAGGGAACAAAGG  R: GGATAAGGCTGGATTATTCTG |
|  |  | c. 316-197C>T | F: GGAGGAAGATAAGAGGTATGAA  R: CACATATTGACCAAATCAGGG |
|  |  | c. 217dup | F: CCTTCCTATGACATGAACTTAACCA  R: GACAGGTACGGCTGTCATCA |
|  |  | c. 180G>C, c. 130G>T, c. 126_129del, c. 85dup, c. 79G>A, c. 68A>C, c.68A>G, c. 52A>T, c. -78A>G, c. -79A>G, c. -100G>A | F: TGAGGTTGTCCAGGTGAG  R: TTGAAGTCCAACTCCTAAGC |
|  | *HBA2* | c. 166del, c. 272_279delAGCTTCGG, c. 427T>C | F: TTCTGGTCCCCACAGACTCA  R: CAAAGACCAGGAAGGGCCG |
|  |  | c. 369C>G | F: CTCTGCACAGCTCCTAAG  R: GAGGTCCTTGGTCTGAGA |
| **qRT-PCR^a^** | *HBA2* | / | F: CCGTGCTGACCTCCAAATACC  R: CCTCCATTGTTGGCACATTCC |
|  | *β-actin* | / | F: TGCTGTCTCCATGTTTGATGTATCT  R: TCTCTGCTCCCCACCTCTAAGT |

^a^qRT-PCR, quantitative Real-time polymerase chain reaction.

**TABLE S2**  The details of high-risk couples

| **Family Number** | **Female** | **Male** | **Pregnant** |
| --- | --- | --- | --- |
| **F01** | -α^3. 7^/αα | --^SEA^/αα | No |
| **F02** | -α^3. 7^/αα | --^SEA^/αα | No |
| **F03** | --^SEA^/αα; HBB c. 126_129del | --^SEA^/αα | Yes |
| **F04** | ααα^anti3. 7^/αα | HBB c. 52A>T | No |
| **F05** | --^SEA^/αα | -α^4. 2^/αα; HBB c. 217dup | Yes |
| **F06** | ααα^anti3. 7^/αα | HBB c. 52A>T | No |
| **F07** | ααα^anti4. 2^/αα; HBB c. 316-197C>T | HBB c. 316-197C>T | Yes |
